# Perceptions of celebrity athletes involved in social cause endorsement

**DOI:** 10.3389/fspor.2024.1415382

**Published:** 2024-10-16

**Authors:** Manuela Biz, Mathias Schubert

**Affiliations:** ^1^ Department of Social Sciences, Media and Sport, Institute of Sport Science, Johannes Gutenberg University, Mainz, Germany; ^2^ Faculty of Movement and Rehabilitation Sciences, KU Leuven, Leuven, Belgium

**Keywords:** athlete brand, cause marketing, brand image, Laureus, ambassador

## Abstract

**Introduction:**

Due to their ability to evoke unique associations in people's minds, professional athletes are considered prime ambassadors for companies, brands or organisations to add intangible values, credibility and awareness to their products or services. Based on that premise, using celebrity athletes for supporting social causes has also become a frequent practice over the past years. Empirical knowledge on how such activities are being perceived by involved athletes is scarce. Little is known about what they consider to be relevant attributes of their personal brand that make them suitable for endorsing social causes.

**Methods:**

Based on semi-structured interviews with (*n* = 12) current and recently retired professional athletes from four different continents, we explore the most prominent attributes of athlete brands as well as other important features in that regard.

**Results:**

Our results indicate that, in contrast to product endorsement, on-field attributes of the athlete brand are considered more important in relation to social causes. On-field attributes, such as career achievements and the behaviour shown during competitions, are seen as the main sources of credibility.

**Discussion:**

Congruence and fit between endorser profiles and cause is perceived as highly relevant for successful cause endorsement activities. Our study is useful for a variety of stakeholders, such as athletes or organisations considering getting involved in social cause endorsement.

## 1 Introduction

Along with the increasing professionalisation and massification of sport, the role of professional athletes has considerably changed (1). Superstar athletes nowadays are popular not only as a consequence of their achievements on the field, but also due to the impact they have on the public by off-field information (2). They demonstrate attributes that lead to an emotional attachment of fans. Based on that, athletes often work as endorsers in different businesses and can become very influential, especially among younger members of society (3–5).

Off-field attitudes and statements from celebrity athletes are also influential to social causes. Even though some critics hold that sports should be apolitical (6), there is a growing list of athletes that use their popularity to support social causes (e.g., Megan Rapinoe, Marcus Rashford). While global sports mega-events, such as the Olympic Games and the FIFA Football World Cup, have always been a vehicle to deliver messages (7), the proliferation of social media increasingly allows and facilitates athletes to speak freely and straight to their fans about their beliefs. In response to the demand for positioning in societal discussions, well-branded athletes are increasingly promoting their philanthropic efforts (8) to guarantee beneficial image protections (6, 7, 9). Being linked to social causes is also an important way of differentiation (8) and an attribute of an athlete's brand (10).

Due to their perceived credibility, athletes have also become very successful ambassadors and endorsers in a great variety of organisations and there is a significant growth in donation intentions to causes once they are supported by athletes (6). There is, however, a lack of empirical knowledge about how such activities are being perceived by involved athletes. In particular, little is known about the relationship between an athlete's personal brand and his/her role as social cause endorser. In order to contribute filling that research gap, the study sought to answer the following research question: What dimensions of an athlete's personal brand are considered relevant and suitable for endorsing social causes by athletes involved? To address this, semi-structured interviews with 12 current and recently retired professional athletes involved in supporting social causes were used to elaborate the attributes they consider relevant in this context.

The study largely builds on a conceptual framework suggested by Hasaan et al. (11), featuring antecedents, on-field and off-field attributes of an athlete's personal brand. Results of the interviews were compared to previous data about the influence of athlete brands in product endorsement contexts.

The paper is structured as follows: In the following two chapters we illustrate the concepts “athlete brand” and “athlete endorsement”, which, together with our theoretical framework presented subsequently, build the conceptual basis of our research. Our methods of data collection and analysis are laid out next. We then present and discuss our results. Practical implications are derived from the key findings and the paper concludes with mentioning limitations of our project as well as directions for future research.

## 2 Literature review and theoretical framework

### 2.1 Athlete brands

A brand may refer to the obtaining of a “certain amount of awareness, reputation and prominence” [(12), p. 7]. The fundamental characteristic of a brand is its differentiation of other offers around, and the main reason to build a brand is to stand out (13). These concepts can be applied to individuals. Consumers are not only attached to traditional brands but also to human brands, which are defined as “any well-known persona who is the subject of marketing communication efforts” [(14), p.104]. Such as traditional brands, athletes are able to evoke unique associations in people's minds as a result of the public recognition of their talents and physical appearance (15). Usually, the athlete is linked to “valued characteristics, such as strength, courage, toughness and power, reflecting integrity, competitiveness and success” [(9), p. 457].

Scholars have been researching athlete brand characteristics (11), the measurement of these brand's equity (10), as well as how fans perceive the personality of the sports celebrity brands (3). Previous studies sustain that the main purpose of establishing an athlete brand is to strengthen the bond between that athlete and fans (11, 16) and that well-branded athletes attract companies seeking effective endorsers (17) and maintain fan support even when performance declines.

Hasaan et al. (11) reinforce Keller's idea (1993) that the creation of an athlete brand starts with awareness. The familiarity with an athlete brand is possible due to different antecedents (11). Based on Keller's brand knowledge scheme (1993), Arai et al. (18) developed the so called “Model of Athlete Brand Image” (MABI) that classified brand attributes into (a) product-related – meaning the elements necessary for performing the product function, and (b) non-product-related, referring to the external aspects related to its consumption. Applying the brand knowledge scheme to an athlete brand, it can be linked to two different dimensions of an athlete's life: performance-related to on-field results as well as behaviour and non-performance related to the off-field lifestyle.

### 2.2 Athlete endorsement

Organisations link themselves to celebrity brands, what can add intangible values, credibility and awareness to their products or services (19). Sports brands and other businesses often count on the great credibility of athletes as supporters or endorsers (3). Due to the attachment of fans, athletes experience a great level of influence among consumers and are very desirable endorsers to brands.

According to Ohanian (20), an endorser has three primary sources of credibility: trustworthiness, perceived attractiveness, and expertise. Trustworthiness refers to the capacity of the celebrity of making himself/herself believable to people, and expertise, how much they dominate. Also, endorsers with a better physical appearance will have a greater chance to influence fans and consumer believes (21). Overall, the more trustworthy and the more attractive a spokesperson is, the better results he/she will have in persuading the public (21, 22). Hoffner and Buchanan (23) state that an endorsement will be better accepted when the public identifies with the spokesperson for having common features, such as gender, race or origin, or due to a wish of being like the endorser (24). Cunningham and Regan (25) have raised the importance of endorser-product fit. Also von Felbert and Breuer (5) highlight the importance of congruence between the endorser and the product or brand. The authors show that the existence of a sports celebrity endorser *per se* does not have a positive direct influence on consumers purchase intentions. Consumers purchase intentions can, however, increase due to a perceived endorser-product congruence.

Previous studies such as Arai et al. (10) consider that an athlete brand has three components that may influence an endorsement: athletic performance (on-field achievements), attractive appearance, and marketable lifestyle (both off-field attributes). According to the authors, the latter has the highest contribution in product endorsement, as it reflects the personality of an athlete. In general, product endorsement is more influenced by the off-field lifestyle of an athlete. Achievements due to athletic performance is only the second most influential element.

Media continuously report about athletes’ off-field activities and the rise of social media contribute to the relevance of lifestyle (8). Also, athletes have more freedom to build their image perceptions when manipulating lifestyle, which is more easily controlled than athletic performance, which depends on a series of factors, such as their opponents’ results (10). Having an attractive appearance, found to be one of the most important factors of credibility in celebrity brands endorsements studies (26), did not show the same effectiveness in athletes’ brands (10).

There is also the factor of fans’ loyalty to be considered. Brands are aware that once fans are loyal to a well-branded athlete, there is a good chance they will also develop a loyal relationship with the athlete's endorsed organisations (3). The admiration the public has for the athlete leads to great influence, as youth and children learn by emulating models. “Sport is a basic social institution that shapes individual and collective models and cultivates social values” [(27), p. 30] and thus it is expected that the athlete takes over this responsibility of spreading the values of the sport to society and the public demands them to show high standards of behaviour (9). Equally, low-performance on-field or controversial attitudes off-field can both easily damage their personal brand and athletes must be aware of it as their image has a fragile nature (28). Also bad behaviour can affect athletes’ brand images and also their credibility as endorsers (18).

Having a positive brand image, which can be explained as the current view people have about a brand (29, 30), depends on building strong and favourable associations (31). Studies influenced by Corporate Social Responsibility (CSR) and Cause Marketing theories show that people have a better perception of brands seen as socially responsible (32, 33) and being associated to social causes is positive also to an athlete brand (8).

Based on these assumptions regarding athlete endorsement in general, the main objective of this research is to provide an academic advance in understanding the endorsement of social causes by looking at it through the lenses of professional (former) athletes involved in such activities. In this paper, celebrity endorsement of social causes in sports is understood as any forms of advocacy by (former) athletes to support social causes by advocating campaigns that aim to raise awareness of issues among the general public [cf. (34, 35)]. We intend to identify what involved athletes consider to be relevant attributes of their personal brand that make them suitable for endorsing social causes. Further, we explore how such dimensions can be compared to those that influence product endorsement, highlighting points in common and in opposition. Thus, the paper aims to deliver information about experiences of social cause endorsement that can be relevant to social projects managers and athletes that wish to engage in this type of partnership.

### 2.3 Theoretical underpinning

The conceptual framework suggested by Hasaan et al. (11) was used as a guiding thread of this analysis. This framework was developed by the authors based on a thorough review of the literature (more than 400 peer-reviewed articles) related to celebrity branding, brand equity, brand loyalty and athlete branding. The search was conducted on two databases (EBSCO, ProQuest) and the Impact Factor of the publication source was take into account for the final selection. In total, 168 studies informed the framework, with publications from the years 1963–2016. Generally, the authors affirm that every brand starts with familiarity (31), meaning that before developing a positive association towards an athlete brand, the fans must be able to identify it and perceive it as different from other athlete brands (36). There is a set of five antecedents that can make an athlete brand identifiable by the public, which are: *Media*; *Oral Communications*; *Impression Management*; *Social Agents*; and *Sport & Team*.

*Media* refers to every sort of communications technology to promote brands to a large public (37) and has a main role in the awareness of an athlete (11, 38, 39). Mass media, including national newspapers, magazines, local and national radio and television and the internet (40), was the first communication channel to athlete brands since the last century and turned athletes into local or global celebrities (41, 42). Nowadays, social media offer to the athletes the opportunity of producing and exchanging content in applications like Instagram, Facebook, or YouTube without a third party's filter (43), strengthening the differentiation of an athlete brand (44). Social media is very popular among sports fans because it allows them to feel closer to their clubs and celebrity athletes (45). Media in this understanding also includes mega-events, such as the Olympic Games or the FIFA Football World Cup, in which athletes experience an intense growth of journalists and sponsors’ interest.

*Oral Communications* refer to informal knowledge spread by the public about a celebrity brand (46). The rumours and narratives combined with facts are elements of an athlete image (47) and building a narrative can generate buzz about an athlete (48). Rumours can impact an athlete brand (49) as the public may trust more on word-of-mouth about a celebrity brand than in media (50). *Impression Management* can be defined as “efforts by an actor to create, maintain, protect, or otherwise alter an image held by a target audience” [(51) p. 1080]. It includes planned changes in gestures, dress, appearance and patterns of talking in order to improve the image projected to the public (52) and is a regular artifice in brand strategies (53), helping to turn athletes into celebrity brands (54). *Social Agents*, such as family members, friends, colleagues and also the community a person belongs to will have a role in the perception of the world (55). The information we get from trusted people about an athlete may influence our perception positively or negatively (56). Last, the awareness of an athlete is directly connected to the relevance of the *Sport* practised as it allows him/her to have more space in the media (57, 58). The *Team* is also relevant as it is highly likely he/she will be admired by the team's supporters and have a negative perception from rival fans (59, 60).

Further to antecedents, considering that an athlete brand is a sum of his/her features and values he/she expresses (61), Hasaan et al. (11) divided the athlete brand in on-field and off-field attributes as a derivation of the product-related and non-product-related brand knowledge scheme (31). That said, athletes become a distinguished brand not only by their on-field achievements but also for what they can show in public off-field activities (47, 62).

On-field attributes are all connected to the performance of an athlete and Hasaan et al. (11) list five different aspects here: athlete's behaviour; team; achievements; style of play; and skills. Being able to exhibit unique skills is an important manner of differentiation from other athletes (63, 64). Fans are also interested in how the athletes behave under pressure, how they connect with teammates and if they exhibit leadership and mental toughness (65). Those who cannot keep focused will be negatively judged by the audience (66). Therefore, people tend to attach themselves to on-field winners (67). Achievements, such as records, trophies, medals and individual prizes are the career's most relevant factors in making an athlete popular (47, 68). To sharpen the focus during the interviews and based on other findings, we merged the different aspects to the concepts of *Behaviour* and *Achievements* in our study (see also Table 2).

Off-field attributes are the set of values a sportsperson will exhibit in moments outside of his/her sporting activity. The bundle in Hasaan et al.'s (11) framework includes physical attraction; lifestyle; personal appeal; ethnicity; and entertainment. The public usually connects athletes with an image of glamour (69), which increases the curiosity about the clothes they wear to their personal relationships (68). Also, in social media, athletes also got a new role as entertainers (70), posting stories about their routines and showing people more of their personality (71). The physical features of a sportsperson are important to fans, both male and female (72–74). It is known that off-field attitudes of the athlete are capable of damaging his/her personal brand (61), but can also be really influential among fans, who may be willing to imitate traces of their idols (10). Again, to narrow down the focus and thus enhance data collection, we reduced the different aspects to *Lifestyle*, *Physical Attraction* and *Ethnicity*.

## 3 Methods

### 3.1 Research design and ethical consent

A qualitative approach was chosen as the information sought after includes predominantly data that is not quantifiable, such as feelings, thoughts and experiences (75). We conducted semi-structured interviews with 12 high-level professional athletes who are either still competing or have recently retired. The study was approved by the research ethics board of the affiliated organisation of the lead author.

### 3.2 Study population and data collection

All interviewees are volunteer ambassadors of Laureus Sports for Good worldwide. According to their own information, Laureus is a global organisation founded in 2000 by two high profile company's (Mercedes-Benz and IWC Schaffhausen) with the objectives of celebrating sports excellence and using it to transform the lives of children and youth in need. The structure of the organisation combines the Laureus World Sports Academy, the Laureus World Sports Awards and its philanthropic wing, the Laureus Sports for Good foundation. The foundation was planned to be a powerful but cost-effective manner to support social causes around the world. Instead of developing the projects themselves, Sports for Good has built a global network of charity structures that have a deeper understanding of local needs. Nowadays, they offer support to over 160 organizations in 40 different countries. One thing that differs Laureus from other organisations is their stake on athletes’ endorsement as the focus of the project since day one. Their work is championed by the Laureus ambassador scheme. Laureus ambassadors are a group of around 235 current or recently retired athletes who have made contributions to the sport and are well recognized by their communities. The ambassadors are distributed all over the world and support the Sport for Good in a more local base by giving their time to raise the credibility and the awareness of a project. They are invited to join the group for their past achievements in sports but also for their relevance in their local community. The group has been successful in the past years in raising funding and getting the public's attention to causes and social projects. That said, all the ambassadors were eligible to be part of the sample as they have a well-recognized image as athletes and are successful as social causes endorsers.

To guarantee diversity in terms of interviewee demographics as well as causes and projects represented, a process of quota sampling was applied. The overall population was divided into categories and volunteers were selected from each bundle in the proportion desired (76). In collaboration with a Laureus officer, we identified three groups that could intensify the diversity of causes and motivations: gender (male or female), disability (able-bodied or disabled) and origin. The last item refers to the assumption that being born in developed or underdeveloped countries affects the degree to which athletes were exposed to social problems or had access to resources through life. Considering these groups, it was established that the interviewees should be at least 40% women, 10% of them should have a disability, and 40% should be natives of underdeveloped countries. Superpositions were accepted.

12 out of 30 athletes contacted accepted the invite. The group fulfilled the sampling exigences and we thus managed to achieve considerable diversity: the group consists of six women, two athletes with a disability and six that were born and competed for/in underdeveloped countries. The interviewees come from seven countries across four continents and are professionals of eight different sports (rugby, cricket, judo, swimming, skating, athletics, cycling, softball). In addition, they have a remarkable athletic background: all of them competed on international level, seven of them were in Olympic or Paralympic Games, four got medals in these events, two hold a record for the number of Olympic and Paralympic medals in their disciplines, five are World Champions and six Continental Champions. Table 1 provides further details about each interviewee and how they fit in the sample. In order to avoid disclosing the interviewees’ identity, it was not possible to mention the sport practised and the personal achievements.

**Table 1:** Set of interviewees and their data.

Athlete	Gender	Origin	Region of residence	Disability	Active	Main causes endorsed
A	Female	Europe	Europe	No	Yes	Gender equity; Mental Health; Sustainability
B	Male	Europe	Europe	No	No	Children's Health; Mental Health
C	Male	South America	South America	Yes	Yes	Disabled People's Rights; Inclusion
D	Female	Europe	Europe	No	Yes	Sports for Refugees as a Tool of Integration; Gender Equity
E	Female	South America	South America	No	No	Sports as a Tool of Development for Children from Poorer Backgrounds; Sports as a Tool of Empowerment for Women
F	Female	North America	North America	No	Yes	Working Mother's Rights; Equal Pay
G	Female	Europe	Europe	No	No	Sports as a Tool of Development for Children from Poorer Backgrounds; Body Positivity for Girls
H	Male	South America	South America	No	Yes	Sports as a Tool of Development for Children from Poorer Backgrounds
I	Male	Africa	Africa	No	No	Sports as a Tool of Development for Children from Poorer Backgrounds
J	Male	Europe	Europe	Yes	Yes	Disabled People's Rights; Inclusion
K	Female	Africa	Africa	No	No	Sports as a Tool of Development for Children from Poorer Backgrounds; Sports as a Tool of Empowerment for Women
L	Male	Africa	Africa/Europe	No	Yes	Racism; Social Entrepreneurship

Due to the geographical spread of the interviewees across the globe, interviews were conducted via video calls in previously scheduled meetings between March and May 2021. The interviews lasted between 40 and 60 min. The interview manual was based on the framework developed by Hasaan et al. (11) as it allowed us to start from already established concepts about athlete brands and to apply them in social cause endorsement. The schedule included questions about antecedents, on-field and off-field attributes of an athlete brand with a particular focus on the context of social cause endorsement (see ch. 2.3). To ensure the questions were approaching the subject in the right way, they were tested with a set of voluntary participants that included native and non-native English speakers. Were considered necessary, rephrasing of questions was carried out jointly within the research team. Questions on the personal background served as ice-breaker questions at the beginning of each interview.

### 3.3 Data analysis

Under consent of all participants, the interviews were recorded. Data was manipulated and analysed following Miles and Huberman's (77) suggestions of procedures, starting with data transcription, what amounted to 160 pages of text. The text was reduced by discarding all irrelevant information, repetitions or meaningless expressions, resulting in 118 pages. During the reduction process, personally identifiable information such as names, countries, sponsors, personal prizes and awards, were also excluded. The files with transcript interviews of each athlete were then identified only by letters (A, B, C, D, E, F, G, H, I, J, K, L), following the order of conducting the interviews. We decided for a deductive coding approach (78) based on the theoretical framework utilized in this research (11). This pre-existing framework served as our “lens” to assess and interpret the data via a “top-down” approach. Thus, our raw data was assigned to the pre-existing themes and their respective codes. The coding system is depicted in Table 2.

**Table 2:** Coding system.

Theme	Code
Antecedents	Media
	Oral communications
	Impression management
	Social agents
	Sport & team
On-field attributes	Behaviour
	Achievements
Off-field attributes	Lifestyle
	Physical attraction
	Ethnicity

Quotes were cropped to exclude information that could lead to the athlete's identification, and received researcher interference in parentheses to ensure comprehension where needed. Quotes were reunited by themes and codes displayed in a sheet. Data was then analysed and the most prominent and frequent opinions among the interviewees were identified. Coding was done manually by one the lead author. In order to ensure reliability in the sense of intersubjective verifiability, the research team frequently met to discuss selected sections of the data (79). During these regular meetings, the research team also discussed whether interpretations and the coding procedures conducted by the lead author were valid and accurate and refinements were taken whenever considered necessary. Relevant information retrieved from the interviewees was then exposed on findings and then compared to current knowledge about athletes’ endorsement in general, in order to identify what is unique to the context of social cause endorsement. All interviewees were offered to review their transcripts after coding, which, however was not claimed by anyone.

## 4 Results and findings

The structure of the following results sections reflects the coding system, i.e., the role of the different antecedents as perceived by the interviewees will be presented first, followed by the respondents’ views on on-field and off-field attributes of their personal brand.

### 4.1 Antecedents

Interviewees highlight the role mass *Media* (in particular, television) had in turning them into known names in their countries. Being proactive in extending the presence on the screen is considered a natural development of their careers, working also as broadcasters, being constantly seen in sports channels in their countries and talking to journalists about causes they support. Interestingly, participants believe media is mainly interested in their on-field activities and not in their private lives. It was also pointed out that there is a difference not only in quantity but also in quality of coverage about women and men in sports, which is seen by them as a cause related to the lower popularity of female athletes among fans.

Interviewees agreed that global events like the Olympic Games or World Championships have a massive impact on one's brand awareness because of the intense interest of the national press and the exposition in sponsors activations:

“The coverage definitely intensifies, especially dealing with an Olympic sport and in [country], because it's the only time that the public really takes notice. And [sport] is not one of the major sports in [country]. It's this two-week period where people really take notice” (Athlete F).

Although the event is momentous, the growth in popularity accompanied the interviewees. It is therefore possible to perceive that the reasons for the popularity of the participants start with how their brands got known by the public. Similar to earlier findings (41, 80, 81), mass media is the main source of the popularity of these athletes and a necessary platform for the athlete to be highly visible. Event live coverage makes athletes popular, even in countries where the sport is not popular (82). Being more present in traditional media reflects on interviewees’ credibility, as the fast spread of online outlets caused scepticism about them and people perceive the statements made in traditional outlets as more reliable (83).

Interviewees also recognize the power of social media in increasing an athlete's awareness in general [cf (84).]. However, some reported low use of social media and pointed to the occurrence of pushback and abuse from the public as the main reason to keep away from it. Some restrict their posts on social media only to their careers, achievements and causes. The main reason for not exposing their private lives is to avoid negative stories that can affect their reputation [cf (85).]. The interviewees that reported a continuous use of social media as a communication tool justified it with two recurrent intentions: engaging with fans and having an open channel to talk about the sport and the causes they support:

“I use my social media as an opportunity to highlight what's happening in the women's game, to share some of the experiences that I'm having with some of my work, and to kind of just highlight that there's loads of opportunities out there for women” (Athlete A).

*Impression Management* tools are commonly applied by body organisations in order to develop the capacity of athletes as spokespersons for the entity. Yet most of the interviewees claim that any improvement on their public persona was not part of a planned strategy, but rather an organic consequence of a maturing process that they went through in the public eye during their careers:

“I never really tried [consciously] to improve anything with my public image. [..] But I think, through the years, I probably got better at doing interviews or appearances and talks just because when you start out as a teenager, you have no idea what you're doing” (Athlete F).

Interviewees state that in many of the social cause opportunities they are involved in, they do so with no vested interests. However, some claim they are better perceived by society since they started doing this job:

“I wouldn't say I gained fans by promoting social causes. But your image as an athlete changes. You become somebody that has all of these attributes that are related to the field and your athletic career but is also somebody that is careful and aware of the causes” (Athlete D).

Our interviews indicate that endorsing products and social causes is mainly seen by the participants as part of their role and something that is expected from them. Although interviewees do not express this intention, the findings support previous information that standing for a social cause can improve an athlete's image (8, 9). However, as a conflicting partnership could affect their image, athletes are careful in their choices. They also claim brands are more open to working with them also in causes:

“I am very proud of the work I have been doing for years together with a few sponsors. (..) I see these companies evolving the HR and hiring people with a disability. I have a lot of freedom to go to these companies and defend this cause with their support” (Athlete C).

All participants say they are truthful in their public appearances, but some of them actively avoid expressing themselves on a few topics in order to prevent negative perceptions from some groups in the population:

“I avoid a lot of subjects and already answering, I don't feel 100% free. I see what we are at a time of great caution in some matters. So, you better not expose that opinion you better keep it to yourself” (Athlete C).

Although they are in this position for their own individual reasons, participants perceive the societal pressure for speaking up on some topics due to being considered as a role model. At this point, they understand that they may be judged if they do not say anything but also if they say something wrong and that both options may affect negatively their image:

“There's a little bit of an insistence on this debate if talking about a cause is mandatory. [..] And it is difficult to judge based on the idea that someone who is very good at something should speak about this other thing” (Athlete H).

Participants believe that rumours and stories about them that circulate among the public, constituting *Oral Communication* about them, are in general positive. Stating that while people do not know all about their private life, participants affirm that what is known by the public is pretty genuine.

“I do think it's close, very close [to the truth]. (…) obviously people don't know everything about me, but I think they get a good idea from what's out there” (Athlete F).

Also the importance of *Social Agents* was confirmed, as interviewees believe that the local prominence of the respective sport is key to gaining awareness towards the general population. There is also a common feeling among them that football receives a level of attention that does not reflect the reality of sports in general. Participants believe that their main link with fans is their passion for a certain *Sport* or a *Team*. Still, there is a relevant attachment based on gender and ethnicity. The idea of the athlete as part of a community is an important source of reliability of the athlete celebrity brand (3). It also relates to the identification model that states a celebrity brand will be more accepted when people recognize common features on the athlete (23, 24).

### 4.2 On-field attributes

Interviewees were able to point to a few traces of their *Behaviour* and skills on-field that pleased their fans and made them stand out from colleagues or competitors. Interviewees, on the stage, focused on winning, not on improving their image. Still, they highlight the need of showing morally strong behaviour to the public, especially values connected to fair play. The athletes also claim that a good performance is a great source of credibility, as people are more open to listening to them when they can back up the speech with good results:

“I think that the achievements bring credibility. You have doors open, let's say to the president of any company if you have an Olympic medal. It is a good business card because it shows your commitment” (Athlete H).

This confirms earlier research that identifies “source credibility” as an important factor in endorsement processes (5). The interviewees reinforce the idea that *Achievements* are not composed just by what they win, but also by other personal records (e.g., being the best of the country), by unprecedented (e.g., the first of the country to be in a final) or even by improbability (e.g., a surprising classification or comeback). The perception of achievements is also influenced by how they reach this peak in their career:

“So not everyone has the physical attributes of Usain Bolt. And even though he has to work extremely hard to win all the medals he did. So, for some people to even just make the Olympics would be an achievement in itself. I think if you give your best, then people appreciate that honesty. And I would have just given them my best that I could have each week” (Athlete J).

Another recurring perception among interviewees is that the public especially appreciates the achievements that result from overcoming difficulties. Some interviewees speak openly about these challenges with the aim that the narrative of their careers is inspiring for their fans:

“I think they like someone that comes from adversity. And throughout my career, I’ve come back from a few injuries, disappointments to become [country] captain winning the [championship]” (Athlete B).

Interviewees believe society has a favourable perception of them for knowing the challenges they surpassed (e.g., injuries and comebacks), and this storytelling can be used to further boost their image (49). It is also believed that having surpassed a difficulty is a meaning source of credibility.

Participants are conscious of the relevance of on-field attributes to their reputation. They can point to particular features that distinguish them from other athletes, as differentiation is a necessary condition to establish an athlete's brand (63). There is a consensus among athletes that the achievements in sport are the most relevant factor in their popularity (47) as fans get more easily attached to successful athletes (67). Athletes say that in competition they show their skills live in front of people with no margin to feint (86), so, in the public perspective, they demonstrate expertise (skills) and trustworthiness, two out of three sources of credibility described by Ohanian (26). The higher weighting of on-field attributes by our interviewees is also due to the fact that performance in competitions is not staged but real (86), what is different to e.g., creating an image in social media. Further, although interviewees have a victorious career, they do not necessarily connect achievements with victories, but with the pursuit of their best and persistence through difficulties (e.g., sexism, disability, pregnancy, injuries, lack of resources). The narratives connect to the archetype of the heroic journey, associated with the role of the athlete since ancient times (87). The hero is someone who stands out from a moral or physical standpoint for realizing an exceptional deed that surpassed his/her possibilities (88).

### 4.3 Off-field attributes

In general, interviewees stated that the focus of their communication efforts is on sport and also on their charity projects, including Laureus events. They try not to show much of their personal *Lifestyle* to the public. The main reasons for this being that they do not see any relevance in doing so or that they want to maintain privacy for themselves and their families. There is, though, some willingness in sharing some information about their personal trips, readings, pets and especially the role as parents:

“I think probably the only thing I really share is that about my dog and I enjoy being outside and that's it. I choose to not share about my boyfriend. Maybe when I do, they [fans] enjoy it because I don't share much, but we give so much when we are on media and that actually it's nice to be able to have some stuff just for me” (Athlete A).

Interviewees tend to avoid sharing negative information about their personal lives (such as break-ups or divorces) and rather share things about parenthood that make them look like ordinary people:

“[Fans] they really like to follow what I do with my kids at home. If my children will be athletes like the father. If I teach them to [sport]” (Athlete C).

Regarding *Physical Attraction*, our interviewees are aware it is an attribute recognized by both male and female fans, but they do not appreciate this type of attention and try actively not to expose their bodies image on off-the-field activities:

“I've chosen not to post pictures of me in a bikini on the beach or training in a sports bra and that type of thing. I definitely haven't used my body as a way to engage with people. I know that that gets more engagement for certain reasons” (Athlete A).

There is a special interest among female interviewees in not reproducing or stimulating stereotypes related to the woman's figure in society in general, such as thinness:

“I was always judged on my body [when competing], so I try to send the message that everybody is ok and I don't promote diets or diet products, for example” (Athlete G).

Off-field attributes are growing in relevance in studies about athlete brands as, due to the intense media coverage, there is a tendency in society to think about athletes in general as pop stars (69). Our interviewees seemed to value the importance of privacy and claim the glamour imagined by people does not apply to their routines. Protecting their privacy is also a manner of image management. It is known that the credibility of an athlete starts on-field, but negative information about off-field activity on media receives great attention from the public and can impose damages to the brand's image (89). Still, in some cases, they expose enough of their routines focusing on common activities, like parenthood, that show similarities with the public. The identification model states that sharing the same experiences with the community makes the athlete more trustworthy (23).

According to previous research, physical attraction also influences the relation between athletes and fans (74) and is a primary dimension of the celebrity's source of credibility (20). Our interviewees consciously avoid benefiting from it, even if this attitude diminishes the influence they have as endorsers. Participants also perceive that physical attraction is only an asset when it fits society's standardized ideal (20). Also referring to the credibility model (ebd., 1991), not exposing off-field problems make them admirable on and off-field, reinforcing the athlete's trustworthiness. The fact they do not expose much of their private lives also keeps the attention to their on-field achievements, their field of expertise and another source of credibility.

Research states that when an athlete share a common characteristic with fans (such as gender, race or ethnicity), he/she is perceived better (90, 91). When asked to identify if their most engaged followers shared features or interests in common with them, the majority of the interviewees pointed that they are mainly the public representative of the respective sport itself. Some athletes claim that they are connected to fans due to their representativity as a person of certain *Ethnicity* in the sport:

“We are known as [place] team. That's not a racial thing. We are a nation full of very colourful people from diverse backgrounds with different thinking. But in the end, we are a team that gives hope and has shown to give opportunity to people from a [place] that hasn't existed in this sport” (Athlete L).

Choosing the right causes to talk about is key for participants: they agree that an ambassador is credible if he/she has a sincere interest in the cause.

“Actually, as I went through my career, I realized that a lot of what I was experiencing in sport as a female was actually replicated and also mirrored in the business world for women around lack of opportunity and growth and support and also visibility” (Athlete A).

When choosing the causes, interviewees claim a sincere interest and connection to the subject as the main source of credibility. We can relate this behaviour to Ohanian's definition of trustworthiness as a source of credibility, as it “refers to the consumer's confidence in the source for providing information in an objective and honest manner” [(20), p. 47]. According to the interviewees, having experienced the social challenges they speak about also guarantees credibility, as they have expertise on the subject. In previous studies about athlete endorsement, having expertise and trustworthiness was found to be the most influential bundle to influence the public's attitude (86). Further, the importance of credibility and also authenticity also reminds us of the importance of a high degree of congruence between an endorser and a cause, as outlined by von Felbert and Breuer (5) as well as Cunningham and Regan (25). Our findings support these assumptions to the extent that certain characteristics of the interviewees are reflected in the selection of causes endorsed (cf. Table 1). Athletes deliberately choose causes that align with their experiences based on e.g., their cultural or geographical background or other characteristics.

The following quote from a disabled athlete who supports causes such as Disabled People's Rights or Inclusion clearly illustrates this.

“If you have lived it, I think it just gives an extra argument that you can draw on these experiences and say, yeah, this thing happened to me. But look, I've come from the other side and this is what can be achieved. And I think that can give people hope for continuing on or trying to better themselves” (Athlete J)

Our interviewees do not see themselves as different but similar to the people in their communities. They understand that they become role models due to this proximity: they are not perfect, but people from the community that are worthy to be emulated because they achieved good things. Participants talked about their focus on surpassing their possibilities and social bias to succeed (e.g., sexism, disability, pregnancy, injuries, lack of resources, lack of support) and believe that these factors increase the admiration they receive from society. In fact, the athlete as a hero is a paramount character (92) and has, for this, the capacity of inspiring fans. Participants acknowledge this and actively publicise the surpassing journey as a manner of inspiring their public to overcome similar bias or difficulties they have in life, This can refer to, for example, a disabled person feeling encouraged to try something new by watching the Paralympics or to a professional woman when facing a male-dominated industry inspired by a female athlete that succeeds in a male-dominated sport.

## 5 Discussion and practical implications

Athletes are valuable spokespersons for both products and social cause organisations. Past research identified athlete characteristics that guarantee their credibility as product endorsers. Our research applied such criteria in the context of social cause endorsement. With a qualitative analysis of data, the study presented the findings obtained from 12 interviews made with high-profile athletes that volunteer as ambassadors in the Laureus Sports for Good Foundation.

In relation to earlier findings about athlete brands and endorsement in general, we can perceive an exchange of priorities between on-field and off-field activities in the context of social cause endorsement. Our interviewees base their image largely on their on-field attributes, whereas earlier research in product endorsement has highlighted the importance of off-field attributes, as this is more easily controllable by athletes in opposition to on-field attributes such as performance and success (10). This is relevant to their reputation (93) and believed to make their endorsement more effective as, for what people know, the ambassadors walk the talk. The relevant professional achievements plus the values expressed during their careers sustain their statements in endorsements. Having athletes with great on-field achievements is also perceived by the interviewees as the main strength in the Laureus project, as the presence of sports idols and celebrities generate the buzz needed to get attention to the causes.

Overall, we perceived consistency in many aspects of an athlete brand: interviewees indicated the importance of expressing the same values on and off the field; conscious of being role models to many, they choose causes connected to their own experiences and work with commercial partners and sponsors that relate to their beliefs. This coherence and congruence in the construction of their image, whether planned or unplanned, is also believed to bring depths to their statements.

Based on our findings, practical recommendations can be derived for stakeholders involved in celebrity endorsement, in particular athletes looking to endorse social causes. Past research has shown that bad behaviour off-field generates negative stories on media and affects the image of the athlete and of the organisation. However, based on our interviews it is plausible to assume that acceptable attitudes off-field do not suffice to make an ambassador effective when supporting a social cause, as the main source of credibility is connected to on-field attributes, such as career achievements and the behaviour shown when competing. Also, organisations may need to look beyond the “big names”. Although global stars have an impressive impact on campaigns, our findings suggest that it can be very effective to pick up as ambassador an athlete well known locally, as the attachment between the athlete and his/her community makes him/her more appealing to that group of persons.

Further, interviewees perceive to be more effective when representing social causes they are authentically interested in and believe that fans are fast to notice when a partnership is made only for financial or public relation purposes. That said, our results indicate the high perceived relevance of endorser-cause congruence, such as a disabled athlete talking about the inclusion of people with a disability or a black athlete talking about racism. However, also athletes that are not directly affected by the social cause they endorse can have good results as ambassadors if they can prove a sincere interest to justify their role (e.g., an athlete with a wealthy family born on a place with a lot of poverty, may have not been directly affected by the lack of resources, but he/she is still part of that community). Also, narratives about resilience of athletes that faced challenges in their careers can be replicated in other people's lives and inspire the public of the organisation in general (e.g., an athlete that surpass a serious injury may be inspiring to anyone who is fighting against a disease). Lastly, interviewees pointed out the insecurity of talking to the press about a subject that they do not master. Therefore, once an athlete is considered a good spokesperson, he/she should be provided with safe and relevant information about the organisation or cause, as an incorrect statement can harm both the organisation's and the athlete's reputation.

## 6 Limitations and scope for future research

This study is the first to discuss perceptions of athletes involved in social cause endorsement and relate this to characteristics of an athlete brand. It thus contributes to a lively (academic) debate on the value and impact of athlete endorsements. The study is, however, not void of some limitations. The first to mention is the lack of calculation of an inter-rater coefficient. While intersubjective verifiability was ensured via frequent consultations of the research team, a coefficient would certainly have increased the quality of the data. Further, the limited set of sample size certainly affects the possibility of drawing generalised results. Also, while restricting our sample to athletes involved in the Laureus ambassador programme made the recruitment of interviewees more feasible due to the support of the organisation, this is of course a considerable delimitation regarding the diversity of our sample. Further, although the study prioritised diversity, none of the twelve participants was or is a professional football player. During the interviews, this fact became a limiting factor, as interviewees pointed out numerous times the gap existent between football and any other sport in most countries. Future qualitative studies should therefore aim for larger samples with more diversity in terms of sports represented (including football). We also hope that our paper has paved the way for quantitative approaches to further investigate social cause endorsement by sport celebrities.

## Data Availability

The raw data supporting the conclusions of this article will be made available by the authors, without undue reservation.

## References

[1] Hasaan A Biscaia R Ross S. Understanding athlete brand life cycle. Sport Soc. (2019) 24:181–205. 10.1080/17430437.2019.1624722

[2] Dix S Phau I Pougnet S. “Bend it like Beckham”: the influence of sports celebrities on young adult consumers. Young Consum. (2010) 11:36–46. 10.1108/17473611011025993

[3] Carlson BD Donavan DT. Human brands in sport: athlete brand personality and identification. J Sport Manag. (2013) 27:193–206. 10.1123/jsm.27.3.193

[4] Derdenger TP Li H Srinivasan K. Firms’ strategic leverage of unplanned exposure and planned advertising: an analysis in the context of celebrity endorsements. J Mark Res. (2018) 55:14–34. 10.1509/jmr.16.0260

[5] Von Felbert A Breuer C. Do endorsements by sports celebrities positively influence consumers’ purchase intentions? Endorser-product congruence and the amplifying influence of consumers involvement in the sport. Int J Sport Manag Mark. (2021) 21:190–208. 10.1504/IJSMM.2021.118811

[6] Galily Y. “Shut up and dribble!”? athletes activism in the age of twittersphere: the case of LeBron James. Technol Soc. (2019) 58:101–9. 10.1016/j.techsoc.2019.01.002

[7] Kim JK Overton H Bhalla N Li JY. Nike, Colin Kaepernick, and the politicization of sports: examining perceived organizational motives and public responses. Public Relat Rev. (2020) 46:101856. 10.1016/j.pubrev.2019.101856

[8] Kunkel T Biscaia R. Sport brands: brand relationships and consumer behavior. Sport Mark Q. (2020) 29:3–17. 10.32731/smq.291.032020.01

[9] Giannoulakis C Drayer J. “Thugs” versus “good guys”: the impact of NBA cares on player image. Eur Sport Manag Q. (2009) 9:453–68. 10.1080/16184740903331796

[10] Arai A Ko YJ Ross S. Branding athletes: exploration and conceptualization of athlete brand image. Sport Manag Rev. (2014) 17:97–106. 10.1016/j.smr.2013.04.003

[11] Hasaan A Kerem K Biscaia R Agyemang KJ. A conceptual framework to understand the creation of athlete brand and its implications. Int J Sport Manag Mark. (2018) 18:169–98. 10.1504/IJSMM.2018.091753

[12] Keller KL. Understanding brands, branding and brand equity. Interact Mark. (2003) 5:7–20. 10.1057/palgrave.im.4340213

[13] Wood L. Brands and brand equity: definition and management. Manag Decis. (2000) 38:662–9. 10.1108/00251740010379100

[14] Thomson M. Human brands: investigating antecedents to consumers’ strong attachments to celebrities. J Mark. (2006) 70:104–19. 10.1509/jmkg.70.3.104

[15] Schouten AP Janssen L Verspaget M. Celebrity vs. influencer endorsements in advertising: the role of identification, credibility, and product-endorser fit. Int J Advert. (2020) 39:258–81. 10.1080/02650487.2019.1634898

[16] Parmentier MA Fischer E. How athletes build their brands. Int J Sport Manag Mark. (2012) 11:106–24. 10.1504/IJSMM.2012.045491

[17] Gladden JM Funk DC. Understanding brand loyalty in professional sport: examining the link between brand associations and brand loyalty. Int J Sports Mark Spons. (2001) 3:54–81. 10.1108/IJSMS-03-01-2001-B006

[18] Arai A Ko YJ Kaplanidou K. Athlete brand image: scale development and model test. Eur Sport Manag Q. (2013) 13:383–403. 10.1080/16184742.2013.811609

[19] Kowalczyk CM Royne MB. The moderating role of celebrity worship on attitudes toward celebrity brand extensions. J Mark Theory Pract. (2013) 21:211–20. 10.2753/MTP1069-6679210206

[20] Ohanian R. The impact of celebrity spokespersons’ perceived image on consumers’ intention to purchase. J Advert Res. (1991) 31:46–54.

[21] Till BD Busler M. Matching products with endorsers: attractiveness versus expertise. J Consum Market. (1998) 15:576–86. 10.1108/07363769810241445

[22] Miciak AR Shanklin WL. Choosing celebrity endorsers. Market Manag. (1994) 3:50–60.

[23] Hoffner C Buchanan M. Young adults’ wishful identification with television characters: the role of perceived similarity and character attributes. Media Psychol. (2005) 7:325–51. 10.1207/S1532785XMEP0704_2

[24] Kamins MA Brand MJ Hoeke SA Moe JC. Two-sided versus one-sided celebrity endorsements: the impact on advertising effectiveness and credibility. J Advert. (1989) 18:4–10. 10.1080/00913367.1989.10673146

[25] Cunningham GB Regan MR. Political activism, racial identity and the commercial endorsement of athletes. Int Rev Sociol Sport. (2011) 47:657–69. 10.1177/101269021141635

[26] Ohanian R. Construction and validation of a scale to measure celebrity endorsers’ perceived expertise, trustworthiness, and attractiveness. J Advert. (1990) 19:39–52. 10.1080/00913367.1990.10673191

[27] Tzachrista V. The Olympic medallist as a transcendental model in antiquity and in the modern age: aspects and approaches. In: Georgiadis K, editor. Olympic Medallists and Olympians as Role Models: Their Educational Role: 4th International Session for Olympic Medallists or Olympians. Athens: International Olympic Academy (2019). p. 27–40.

[28] Ilicic J Webster CM. Consumer values of corporate and celebrity brand associations. Qual Mark Res. (2015) 18:164–87. 10.1108/QMR-06-2013-0037

[29] Herzog H. Behavioral science concepts for analyzing the consumer. In: Bliss P, editor. Marketing and the Behavioral Sciences. Boston: Allyn and Bacon, Inc. (1963). p. 76–86.

[30] Newman JW. New insight, new progress, for marketing. Harv Bus Rev. (1957) 35:95–102.

[31] Keller KL. Conceptualizing, measuring, and managing customer-based brand equity. J Mark. (1993) 57:1–22. 10.1177/002224299305700101

[32] Brown TJ Dacin PA. The company and the product: corporate associations and consumer product responses. J Mark. (1997) 61:68–84. 10.1177/002224299706100106

[33] Sen S Bhattacharya CB. Does doing good always lead to doing better? Consumer reactions to corporate social responsibility. J Mark Res. (2001) 38:225–43. 10.1509/jmkr.38.2.225.18838

[34] del Mar García de los Salmones M Dominguez R. Celebrity endorsement and involvement with the social cause in nonprofit organizations. J Nonprofit Public Sect Mark. (2016) 28:309–26. 10.1080/10495142.2016.1237922

[35] Van den Bulck H Panis K Van Aelst P Hardy A. Celebrity activists in social profit campaigning: a survey with the flemish public on views and effectiveness. Annual Conference of the International Communication Association, Singapore (2010).

[36] Biscaia R Correia A Rosado AF Ross SD Maroco J. Sport sponsorship: the relationship between team loyalty, sponsorship awareness, attitude toward the sponsor, and purchase intentions. J Sport Manag. (2013) 27:288–302. 10.1123/jsm.27.4.288

[37] O'Keeffe M Zawadzka J. Does passion for a team translate into sales for a sponsor? The Irish case. J Spons. (2011) 4:190–6.

[38] Rindova VP Pollock TG Hayward ML. Celebrity firms: the social construction of market popularity. Acad Manag Rev. (2006) 31:50–71. 10.5465/amr.2006.19379624

[39] Vincent J Hill JS Lee JW. The multiple brand personalities of David Beckham: a case study of the Beckham brand. Sport Market Q. (2009) 18:173–80.

[40] Boyle R Haynes R. Football in the New Media Age. London: Routledge (2004).

[41] Liu MT Brock JL. Selecting a female athlete endorser in China: the effect of attractiveness, match-up, and consumer gender difference. Eur J Mark. (2011) 45:1214–35. 10.1108/03090561111137688

[42] Summers J Morgan MJ. More than just the media: considering the role of public relations in the creation of sporting celebrity and the management of fan expectations. Public Relat Rev. (2008) 34:176–82. 10.1016/j.pubrev.2008.03.014

[43] Kaplan AM Haenlein M. Two hearts in three-quarter time: how to waltz the social media/viral marketing dance. Bus Horiz. (2011) 54:253–63. 10.1016/j.bushor.2011.01.006

[44] Lebel K Danylchuk K. Facing off on twitter: a generation Y interpretation of professional athlete profile pictures. Int J Sport Commun. (2014) 7:317–36. 10.1123/IJSC.2014-0004

[45] Sierra JJ Taute HA Heiser RS. Personal opinions and beliefs as determinants of collegiate football consumption for revered and hated teams. Sport Market Q. (2010) 19:143–53. 10.1007/978-3-319-11797-3_53

[46] Künzler D Poli R. The African footballer as visual object and figure of success: Didier Drogba and social meaning. Soccer Soc. (2012) 13:207–21. 10.1080/14660970.2012.640502

[47] Grant N Heere B Dickson G. New sport teams and the development of brand community. Eur Sport Manag Q. (2011) 11:35–54. 10.1080/16184742.2010.537364

[48] Crawford G. Cultural tourists and cultural trends: commercialization and the coming of the storm. Sport Soc. (2002) 5:21–38.

[49] Carter D. Money Games: Profiting from the Convergence of Sports and Entertainment. Redwood City, CA: Stanford University Press (2010).

[50] Rosen E. The Anatomy of Buzz: How to Create Word-of-Mouth Marketing. New York: Doubleday (2000).

[51] Bolino MC Kacmar KM Turnley WH Gilstrap JB. A multi-level review of impression management motives and behaviors. J Manag. (2008) 34:1080–109. 10.1177/0149206308324325

[52] James MS. Female sports celebrities targeting female teenagers: a content analysis of magazine advertising. J Bus Econ Res. (2010) 8:1–13. 10.19030/jber.v8i1.653

[53] Agyemang KJ Williams AS. Creating revenue via organisational “brandpression” management (ObpM): a marriage of brand management and impression management in professional sport. Int J Rev Manag. (2013) 7:171–81. 10.1504/IJRM.2013.055687

[54] Wagg S. Angels of US all? Football management, globalization and the politics of celebrity. Soccer Soc. (2007) 8:440–58. 10.1080/14660970701440725

[55] Dixon K. Learning the game: football fandom culture and the origins of practice. Int Rev Sociol Sport. (2013) 48:334–48. 10.1177/1012690212441157

[56] Bush AJ Martin CA Bush VD. Sports celebrity influence on the behavioral intentions of generation Y. J Advert Res. (2004) 44:108–18. 10.1017/S0021849904040206

[57] Manzenreiter W Horne J. Playing the post-fordist game in/to the far east: the footballisation of China, Japan and South Korea. Soccer Soc. (2007) 8:561–77. 10.1080/14660970701440899

[58] Richelieu A. The internationalisation of a sports team brand: the case of European soccer teams. Int J Sports Mark Spons. (2008) 3:10–22. 10.1108/IJSMS-10-01-2008-B006

[59] Robinson MJ Trail GT. Relationships among spectator gender, motives, points of attachment, and sport preference. J Sport Manag. (2005) 19:58–80. 10.1123/jsm.19.1.58

[60] Heere B James J Yoshida M Scremin G. The effect of associated group identities on team identity. J Sport Manag. (2011) 25:606–21. 10.1123/jsm.25.6.606

[61] Cortsen K. Annika Sörenstam—a hybrid personal sports brand. Sport Bus Manag. (2013) 3:37–62. 10.1108/20426781311316898

[62] Wu SH Tsai CYD Hung CC. Toward team or player? How trust, vicarious achievement motive, and identification affect fan loyalty. J Sport Manag. (2012) 26:177–91. 10.1123/jsm.26.2.177

[63] Abernethy B. Anticipation in squash: differences in advance cue utilization between expert and novice players. J Sports Sci. (1990) 25:17–34. 10.1080/02640419008732128 2359149

[64] Theysohn S Hinz O Nosworthy S Kirchner M. Official supporters clubs: the untapped potential of fan loyalty. Int J Sports Mark Spons. (2009) 10:33–55. 10.1108/IJSMS-10-04-2009-B004

[65] Koernig SK Boyd TC. To catch a tiger or let him go: the match-up effect and athlete endorsers for sport and non-sport brands. Sport Market Q. (2009) 18:25.

[66] Lear KE Runyan RC Whitaker WH. Sports celebrity endorsements in retail products advertising. Int J Retail Distrib Manag. (2009) 27:308–21. 10.1108/09590550910948547

[67] Kaynak E Salman GG Tatoglu E. An integrative framework linking brand associations and brand loyalty in professional sports. J Brand Manag. (2008) 15:336–57. 10.1057/palgrave.bm.2550117

[68] Chadwick S Burton N. From Beckham to Ronaldo—assessing the nature of football player brands. J Spons. (2008) 1:307–17.

[69] Woodhouse D Williams J. Offside? the Position of Women in Football. London: Garnet (1999).

[70] Andrews DL Jackson SJ. Introduction: sport celebrities, public culture, and private experience. In: Andrews DL Jackson SJ, editors. Sport Stars: The Cultural Politics of Sporting Celebrity. London: Routledge (2001). p. 1–19.

[71] Larson E Larson R. The Influencing Formula. Minneanapolis: Watermark Learning Publications (2012).

[72] Hogan MJ Strasburger VC. Body image, eating disorders, and the media. Adolesct Med. (2008) 19:521–46. 10.1542/9781581104103-body 19227390

[73] Pope S. “Like pulling down Durham cathedral and building a brothel”: women as “new consumer” fans? Int Rev Sociol Sport. (2011) 46:471–87. 10.1177/1012690210384652

[74] Van Amsterdam N Knoppers A Claringbould I Jongmans M. A picture is worth a thousand words: constructing (non-) athletic bodies. J Youth Stud. (2012) 15:293–309. 10.1080/13676261.2011.643233

[75] Gratton C Jones I. Research Methods for Sports Studies. London: Routledge (2014).

[76] Acharya AS Prakash A Saxena P Nigam A. Sampling: why and how of it. Indian J Med Spec. (2013) 4:330–3. 10.7713/ijms.2013.0032

[77] Miles MB Huberman AM. Qualitative Data Analysis: An Expanded Sourcebook. Thousand Oaks, CA: Sage (1994).

[78] Kuckartz U Rädiker S. Analyzing Qualitative Data with MAXQDA. Basel: Springer International Publishing (2019).

[79] Guest G MacQueen KM Namey EE. Applied Thematic Analysis. Thousand Oaks: Sage (2012).

[80] O'Reilly NJ Braedley LA. Celebrity athletes and athletic clothing design: branding female tennis players. Int J Sport Manag Mark. (2008) 3:119–39. 10.1504/IJSMM.2008.015964

[81] Hu AWL Tang LR. Factors motivating sports broadcast viewership with fan identification as a mediator. Soc Behav Personal. (2010) 38:681–9. 10.2224/sbp.2010.38.5.681

[82] Koenigstorfer J Groeppel-Klein A Kunkel T. The attractiveness of national and international football leagues: perspectives of fans of “star clubs” and “underdogs”. Eur Sport Manag Q. (2010) 10:127–63. 10.1080/16184740903563406

[83] Salaudeen MA Onyechi N. Digital media vs mainstream media: exploring the influences of media exposure and information preference as correlates of media credibility. Cogent Arts Human. (2020) 7:1837461. 10.1080/23311983.2020.1837461

[84] Pronschinske M Groza MD Walker M. Attracting Facebook “fans”: the importance of authenticity and engagement as a social networking strategy for professional sport teams. Sport Market Q. (2012) 21:221–31.

[85] Shakib S Veliz P. Race, sport and social support: a comparison between African American and white youths’ perceptions of social support for sport participation. Int Rev Sociol Sport. (2013) 48:295–317. 10.1177/1012690212439172

[86] Koo GY Ruihley BJ Dittmore SW. Impact of perceived on-field performance on sport celebrity source credibility. Sport Market Q. (2012) 21:147–58.

[87] Rubio K Veloso R Leao L. Between solar and lunar hero: a cartographic study of Brazilian Olympic athletes in the social imaginary. Imago. (2018) 11:147–62. 10.7413/22818138118

[88] Campbell J. O Poder do Mito. Sao Paolo: Palas Athena (1990).

[89] Money RB Shimp TA Sakano T. Celebrity endorsements in Japan and the United States: is negative information all that harmful? J Advert Res. (2006) 46:113–23. 10.2501/S0021849906060120

[90] Fletcher T. “Who do “they” cheer for?” cricket, diaspora, hybridity and divided loyalties amongst British Asians. Int Rev Sociol Sport. (2011) 47:612–31. 10.1177/1012690211416556

[91] Wong CJ. Boundaries of Obligation in American Politics: Geographic, National, and Racial Communities. Cambridge: Cambridge University Press (2010).

[92] Rubio K. The mythic relation between the athlete and the hero: the contemporary sport imaginary. In: Georgiadis K, editor. Olympic Medallists and Olympians as Role Models: Their Educational Role: 4th International Session for Olympic Medallists or Olympians. Athens: International Olympic Academy (2019). p. 61–7.

[93] Daley C Wolfson S. Leadership at distance: English football fans’ trust in Sven Goran Eriksson and David Beckham during the 2006 world cup. Sport Exerc Psychol Rev. (2010) 6:3–18. 10.53841/bpssepr.2010.6.1.3

